# Effectiveness of MF59™ Adjuvanted Influenza A(H1N1)pdm09 Vaccine in Risk Groups in the Netherlands

**DOI:** 10.1371/journal.pone.0063156

**Published:** 2013-04-30

**Authors:** Leonoor Wijnans, Jeanne Dieleman, Bettie Voordouw, Miriam Sturkenboom

**Affiliations:** 1 Erasmus Medical Center, Rotterdam, The Netherlands; 2 Medicines Evaluation Board, Utrecht, The Netherlands; University of Hong Kong, Hong Kong

## Abstract

**Background:**

The aim of the present study was to estimate the effectiveness of the MF59™-adjuvanted influenza A(H1N1)pdm09 vaccine against medically attended influenza-like illness and RT-PCR confirmed influenza in the at-risk population and persons over 60 in the Netherlands.

**Methods:**

We conducted a retrospective cohort study in a Dutch based GP medical record database between 30 November 2009 and 1 March 2010 to estimate the vaccine effectiveness against influenza-like illness. Within the cohort we nested a test negative case-control study to estimate the effectiveness against laboratory confirmed influenza.

**Results:**

The crude effectiveness in preventing diagnosed or possible influenza-like illness was 17.3% (95%CI: −8.5%–36.9%). Of the measured covariates, age, the severity of disease and health seeking behaviour through devised proxies confounded the association between vaccination and influenza-like illness. The adjusted vaccine effectiveness was 20.8% (95%CI: −5.4%, 40.5%) and varied significantly by age, being highest in adults up to 50 years (59%, 95%CI: 23%, 78%), and non-detectable in adults over 50 years. The number of cases in the nested case control study was too limited to validly estimate the VE against confirmed influenza.

**Conclusions:**

With our study we demonstrated that the approach of combining a cohort study in a primary health care database with field sampling is a feasible and useful option to monitor VE of influenza vaccines in the future.

## Introduction

Vaccination is regarded as one of the most efficient interventions that protect the population at risk of serious health complications during influenza pandemics [1]. During the H1N1-influenza pandemic of 2009/2010 mass vaccination campaigns with new influenza vaccines were set out throughout the world. In order to reduce the amount of antigen needed for vaccinating entire populations oil-in-water adjuvanted vaccines were used for the first time on a large scale in Europe [2]. In the Netherlands general practitioners (GPs) were provided with MF59™-adjuvanted influenza A(H1N1)pdm09 vaccines [3] to vaccinate persons at risk due to underlying comorbidities and persons over 60 years of age. These persons were offered two doses of the vaccine.

The MF59-adjuvanted influenza A(H1N1)pdm09 vaccine was licensed based on immunogenicity and safety of vaccines with avian influenza strains, allowing for fast track roll out of vaccines upon the emerging pandemic [2]. Estimates of the effectiveness of the vaccine in targeted risk groups are scarce to date [4], [5], [6]. Steens *et al* reported no significant vaccine effectiveness (VE) (19%, 95%CI: −28, 49) against influenza A(H1N1)pdm09 -infection related hospitalisation in a matched case control study in targeted risk groups in the Netherlands [4]. Castilla *et al* [5] conducted a cohort study in all non-institutionalized persons in a region in Spain where children (1–17 years) and persons aged over 60 received the MF59™-adjuvanted influenza A(H1N1)pdm09 vaccine. They found no evidence of effectiveness of vaccination against medically attended influenza-like illness (ILI) in children (VE: 12%; 95%CI: −142%, 68%) and in the elderly (VE: 25%; 95%CI: −19%, 53%).

Data on effectiveness of vaccination programmes with adjuvanted vaccines in different target groups is essential to inform future decisions and recommendations for vaccination programmes and possible complementary or alternative public health measures in order to mitigate the potential impact of influenza epidemics and pandemics. The aim of our study was to estimate the effectiveness of the MF59™-adjuvanted influenza A(H1N1)pdm09 vaccine against medically attended ILI and against laboratory confirmed A(H1N1)pdm09 infection in the population that was indicated for vaccination by the GP in the Netherlands.

## Methods

We conducted a retrospective cohort study in a Dutch GP medical record database, in which we nested a case control study to determine effectiveness of the MF59™-adjuvanted influenza A(H1N1)pdm09 vaccine against RT-PCR confirmed influenza infection.

### IPCI Database

Our cohort was identified within the Integrated Primary Care Information (IPCI) database. More detailed information on IPCI has been published elsewhere [7]. In short, IPCI contains longitudinal data from anonymized computer-based medical records of Dutch GPs from 1996 onwards. In the Netherlands, almost all residents are registered with a GP or practice, which serves as the gatekeeper to and from all medical care in the Netherlands. The age and gender distribution of the population in IPCI is representative of the Netherlands and of community dwelling persons. Currently, IPCI contains information on over 1,100,000 patients from over 200 participating GP practices located throughout the Netherlands. IPCI includes anonymous demographic information as well as information on signs, symptoms and diagnoses, both coded through the International classification of primary care (ICPC) and as free text, prescriptions (ATC coded), annual vaccinations against influenza and non-childhood vaccines, hospital admissions, referrals to secondary care, letters from specialists, and laboratory test results. Records have good validity for prescriptions, hospitalizations, influenza vaccination and influenza related outcomes [7], [8], [9]. The IPCI database complies with European Union guidelines on the use of medical data for medical research [7]. Approval for this study was obtained by the Scientific and Ethical Advisory Board of the IPCI project and by the Medical Ethical committee of Erasmus MC. Informed consent was obtained from all patients participating in the nested case control study.

### Study Population

#### Cohort

We defined a cohort within the IPCI database of persons who were eligible for A(H1N1)pdm09 vaccination through the GP due to an underlying medical condition or age >60 years and who had at least one year of valid database history. As pregnancy is not consistently recorded from the start of pregnancy, only pregnant women indicated for A(H1N1)pdm09 vaccination due to underlying medical conditions were included in the cohort. Eligibility for vaccination was assessed from the electronic patient records using free text and ICPC-code searches followed by manual verification in the full electronic medical record.

We excluded GPs with incomplete or unreliable registration of vaccination defined as a coverage of influenza A(H1N1)pdm09 vaccine in persons >60 years lower than 50%, or with unreliable vaccination dates. In addition, we excluded persons with a contraindication to influenza vaccination and persons who had visited the GP for ILI between start of circulation of H1N1 in the Netherlands (week 28) and start of follow-up (week 49).

#### Nested case control

Practices included in the cohort study were invited to participate in the case control study. Cases and controls were obtained from cohort members who visited the GP for ILI during the study period. Controls were to be matched to cases by GP practice and time of presentation.

### Study Period

#### Cohort

Follow-up started on 30 November 2009 (week 49), two weeks after the majority of GP practices had administered the 1^st^ dose. Follow-up ended at death, first ILI, transferring out of the practice, or end of the study period (1 March 2010).

#### Nested case control

The swab schedule for the nested case control study was planned to start two weeks after start vaccination as indicated by participating GPs. Swabbing started on 9 November 2009 and ended on 3 March 2010.

### Study Endpoint

#### Cohort

The outcome of interest was medically attended ILI using the European ILI case definition [10]: a sudden onset of symptoms combined with 1) at least one of the following symptoms: fever or feverishness, malaise, headache, or myalgia; and 2) at least one of the following three respiratory symptoms: cough, sore throat, shortness of breath.

ILI cases were extracted from the IPCI database by using an extensive string search including free text terms combined with ICPC-codes (R80, R81, R74, R78) reflecting the symptoms and diagnosis of ILI. Obvious negations were excluded. All identified ILI cases from week 30 onwards were manually validated against the full electronic patient record to check whether they met the case definition, validation was done while being blinded to exposure.

#### Nested case control

The primary outcome in the case control study was RT-PCR confirmed influenza in persons presenting to the GP with ILI. A nasopharyngeal swab was taken from cohort members with ILI symptoms during the influenza season. Nasopharyngeal swabs were sent to the virology department of the Erasmus-MC for RT-PCR analysis. All persons with samples tested positive for influenza infection were classified as cases. Cases were sub-typed as influenza A(H1N1)pdm09, H1N1, H3N2 or B. Persons with ILI but no detectable influenza were classified as controls.

### Exposures

The primary exposure of interest in this study was vaccination with MF59™-adjuvanted influenza A(H1N1)pdm09 vaccine. Persons having received at least a first dose of vaccine at the start of follow-up (cohort) or at time of swabbing (nested case control) were considered exposed, regardless of the time since vaccination. Vaccination status was determined through GP-specific free text searches and ICPC-codes in the full electronic patient record followed by random manual verification to assess and increase the specificity of the final search. Distinction between seasonal influenza vaccination and doses of H1N1-vaccinations were based on free text wording and calendar dates. Information on the following covariates at baseline was collected from the electronic patient record for each individual in the cohort: age, gender, presence of co-morbidity (diabetes, respiratory, cardiovascular, renal insufficiencies, immune-compromised or malignancies; identified through free text searches and ICPC-codes followed by manual verification against the electronic records), seasonal influenza vaccination history, use of oseltamivir, zanamivir, amantadine, rimantadine, health care utilization (defined as number of GP-visits in previous year) and severity of underlying comorbidity (estimated by the number of different drugs prescribed in previous year identified by number of different ATC-codes).

Participants in the nested case control study had a unique study ID that was linked to their unique patient identifier in the IPCI database. Information on exposure and covariates was extracted from the IPCI-database.

### Statistical Methods

#### Cohort

Descriptive analyses and univariate analysis were performed to compare study population baseline characteristics between vaccinated and unvaccinated patients. We estimated crude and adjusted estimates for VE (1-relative risk*100%) for ILI through univariate and multivariate Cox-proportional hazard analysis. We used subject time, which was calendar time, as the time axis. Variables were included in the multivariate analysis if they changed the crude point-estimate by more than 10%.

#### Nested case control

Crude odds ratios with 95% confidence intervals were obtained by using conditional logistic regression analysis. The crude VE was computed as VE = 1– OR.

#### Sensitivity analyses

In the cohort, misclassification of exposure was investigated by varying the start of the follow-up period (starting at week 47 and week 51 instead of 49), and varying the definition of exposure. In this analysis persons were considered exposed if they were vaccinated >14 days prior to baseline or >7 days prior to baseline. All other persons were considered unexposed. Additionally, we conducted a post hoc analysis in which vaccination was considered as a time dependent variable, meaning the exposure status was determined when an outcome occurred. Persons were considered exposed 14 days after vaccination. In this analysis baseline could be brought back to 01-10-2009, which increased the number of cases. As vaccination was time dependent misclassification was also minimized.

Statistical significance was accepted at a p-value <0.05. All analyses were done using SPSS (SPSS Inc., Chicago, IL, USA) version 15.0 for Windows.

## Results

### Study Population

#### Cohort

At the start of follow-up there were 191,518 persons who had an indication for influenza A(H1N1)pdm09 vaccination in 205 GP practices contributing data to IPCI. Of these, 68,642 persons from 102 GP practices were excluded, as influenza A(H1N1)pdm09 vaccination could not be assessed reliably in the electronic patient record. Of the remaining 122,876 persons, 1,430 had ILI between week 28 and start of follow-up (week 49) and were excluded as they were not at risk of H1N1 ILI anymore (assuming infection with H1N1). The final study population for the primary analysis included 121,446 patients with an average follow-up time of 75.8 days per person (SD 22.2) from week 49 onwards.

#### Nested case control

In total, 41 GP practices agreed to participate in the nested case control study. Two dropped out early due to time constraints.

### Baseline Characteristics Cohort

The A(H1N1)pdm09 vaccinated and non-vaccinated persons differed regarding a number of baseline characteristics (Table 1). Unvaccinated persons were younger and less likely to have received a seasonal influenza vaccine in 2008 and 2009. The majority of the cohort (73.5%) had at least one type of underlying disease that would qualify as indication for vaccination, thus including healthy people 60 years or older. With the exception of diabetes and respiratory disease, co-morbidities were more prevalent in vaccinated as compared to unvaccinated persons, most notably for cardiac disease and malignancies. The mean number of different drugs prescribed in the preceding year was higher in vaccinated persons, as was the number of GP contacts in the preceding year.

**Table 1:** Baseline characteristics cohort.

	*Exposed to first dose influenza A(H1N1)pdm09 vaccine* 6					
		unexposed		Exposed		
		*51442*		*70004*		
		*n*	*(%)*	*n*	*(%)*	*p-value*
**Age** 1	*Mean (st.dev)*	49.8	(22.5)	63.6	(16.8)	<0.0001
	*< = 4*	938	(1.8)	371	(0.5)	
	*5–19*	6717	(13.1)	2445	(3.5)	
	*20–49*	14266	(27.7)	7216	(10.3)	
	*50–59*	7223	(14.0)	7315	(10.4)	
	*60–79*	18809	(36.6)	43235	(61.8)	
	*80+*	3489	(6.8)	9422	(13.5)	
**Gender**	*male*	24720	(48.1)	32290	(46.1)	<0.0001
**Seasonal influenza vaccination 2009**		12744	(24.8)	59965	(85.7)	<0.0001
**Seasonal influenza vaccination 2008**		15153	(29.5)	51522	(73.6)	<0.0001
**Number of pandemic H1N1 vaccine doses on 30-11-09**	*None*	51442	(100)			
	*1 dose*			67048	(95.8)	
	*2 doses*			2956	(4.2)	
**Mean number of days since first dose (SD)**						
**Days since first dose on 30-11-2009**	<7			11568	(16.5)	
	7–14			31420	(44.9)	
	≥14			35494	(50.7)	
**Diabetes**		16063	(31.2)	16269	(23.2)	<0.0001
**Cardiac disease**		12752	(24.8)	32781	(46,8)	<0.0001
**Respiratory disease**		12208	(23.7)	18840	(26,9)	<0.0001
**Renal disease**		884	(1.7)	2218	(3.2)	<0.0001
**Malignancy**		4929	(9.6)	10717	(15.3)	<0.0001
**Immune compromised**		95	(0.2)	199	(0.3)	<0.0001
**Any chronic co-morbidity** 3		36334	(70.6)	53012	(75.7)	<0.0001
**Mean number of different drugs prescribed** 2,4	*Mean (st.dev)*	3.69	(4.1)	6.0	(4.9)	<0.0001
**Mean number of GP contacts** 2	*Mean (st.dev)*	11.0	(11.6)	17.3	(13.2)	<0.0001
**Use of antiviral drugs** 5 **before 30-11-2009**		130	(0.3)	246	(0.4)	0.002
**Use of antiviral drugs** 5 **after 30-11-2009**		13	(0.0)	38	(0.1)	0.015

1 On 30-11-2009.

2 Between 01-10-2008 and 01-10-2009.

3 Includes respiratory, cardiovascular, diabetes and renal disease, persons with malignancies and immune compromised.

4 Based on ATC (7 digits).

5 Antiviral drugs: Amantadine, rimantadine, oseltamivir, zanamivir which are all indicated for treatment of influenza infection; amantadine is also used in the treatment of parkinsons disease.

6 In the analyses those with ILI prior to start of follow-up (30-11-09) were excluded.

### Vaccination

**Figure 1:**
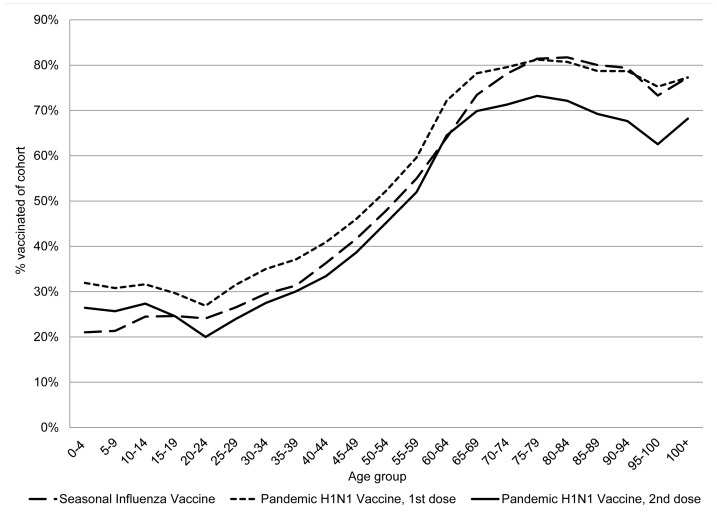
Vaccination coverage per age group. Vaccination coverage per age group for seasonal influenza vaccination and first and second doses of influenza A(H1N1)pdm09 vaccine in the cohort of patients that had an indication for pandemic influenza vaccination.

**Figure 2:**
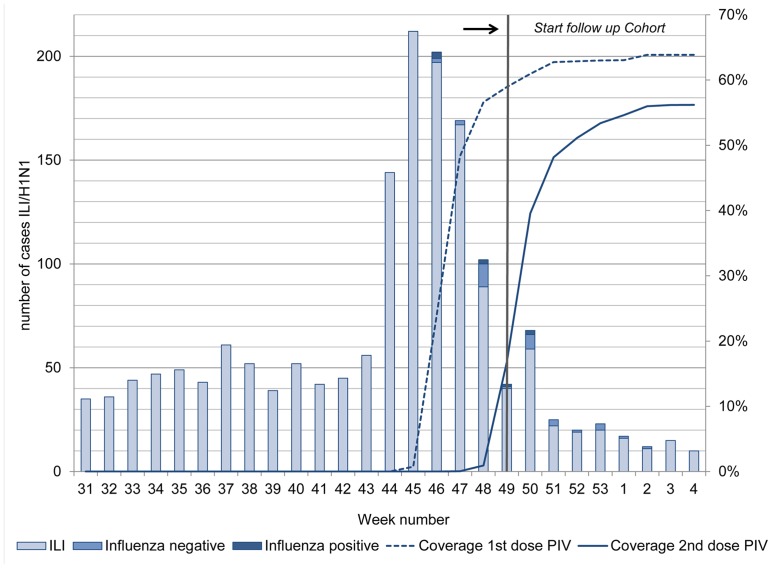
Influenza vaccination in relation to the pandemic curve. Coverage of the 1^st^ and 2^nd^ dose of the influenza A(H1N1)pdm09 vaccine, ILI cases in the cohort, and influenza positive cases plus controls (influenza negative) from the nested case control study against calendar time.

Vaccine uptake was highest in persons 60 years and older (Figure 1). By the end of the vaccination campaign, 88% of those having received a first dose also received a second dose of the influenza A(H1N1)pdm09 vaccine. At the start of follow-up, which was before the end of the vaccination campaign, point coverage for seasonal influenza vaccination in the cohort was 59.8%. For a single dose of influenza A(H1N1)pdm09 vaccine it was 57.6% and for two doses it was 4.2% (Figure 2). Fifty-one % of vaccinated persons had received a first dose at least 14 days before the start of the study. Only 16% had received their first dose less than 7 days before the start of the study.

### ILI & RT-PCR Confirmed Influenza

In the total cohort, 255 ILI cases were identified during follow up. The incidence rate of ILI during follow-up was age dependent, being highest in the youngest age group and slightly lower in subsequent age groups (Table 2). The overall incidence rate during follow-up was 10.1 per 1000 person years (95%CI: 8.9–11.4).

**Table 2:** Number and rate of influenza like illness (ILI) cases.

	*Age category*					
	< = 4	5–19	20–49	50–59	60–79	80+
**Number of ILI cases (%)**	4 (0.31%)	24 (0.26%)	59 (0.27%)	29 (0.20%)	112 (0.18%)	27 (0.21%)
**Person Time** 1	268	1839	4319	2982	13106	2714
**Incidence Rate (95%CI)** 2	14.9 (5.60–39.73)	13.1 (8.75–19.47)	13.7 (10.58–17.63)	9.7 (6.76–13.99)	8.6 (7.10–10.28)	9.9 (6.82–14.50)

1 In years.

2 per 1,000 person- years.

Figure 2 shows the distribution of validated ILI cases, influenza positive and negative cases from the case control study over calendar time along with the coverage of the first and second dose of pandemic influenza vaccination. Vaccination started around the peak of ILI incidence.

In the nested case control study 46 swabs were received for analysis. One swab could not be analysed. Of the remaining 45, 9 tested positive for influenza A, including 7 confirmed A(H1N1)pdm09 infections. The average age of cases was 33.4 years (SD: 22.3 years), controls were older with an average age of 55.4 years (SD 20.5 years).

### Vaccine Effectiveness

In the total cohort, we found a crude VE estimate against ILI of 17.3% (95%CI: −8.5%, 36.9%). Of the measured covariates, age, the number of different drugs prescribed in the preceding year and the number of GP contacts in the preceding year confounded the association between influenza A(H1N1)pdm09 vaccine and ILI with at least a 10% change in the point estimate. The adjusted VE against ILI was 20.8% (95%CI: −5.4%, 40.5%) (table 3). The VE differed by age groups, with the highest adjusted VE in adults up to 50 years (59%, 95%CI: 23%,78%).

**Table 3:** Crude and adjusted pandemic H1N1 vaccine effectiveness per age category: Primary analysis with baseline at 30-11-2009 & Post hoc Time Dependent Analysis with baseline at 01-10-2009.

*Age Category*	*Number of ILI cases (%)*	*Crude VE*	*95%CI*	Adjusted VE*	*95%CI*
**Overall**		17.3%	−8.5% to 36.9%	20.8%	−5.4% to 40.5%
**< = 4 yrs**	4 (0.31%)	−482.9%	−6988.3% to 52.1%	−505.8%	−8341.8% to 56.5%
**5–19 yrs**	24 (0.26%)	38.7%	−85.4% to 79.8%	50.9%	−51.0% to 84.0%
**20–49 yrs**	59 (0.27%)	42.2%	−7.1% to 68.8%	58.7%	22.7% to 77.9%
**50–59 yrs**	29 (0.20%)	17.7%	−79.4% to 62.3%	20.9%	−76.1% to 64.5%
**60–79 yrs**	112 (0.18%)	−36%	−122% to 16%	−14,2%	−86.7% to 30.1%
**80+yrs**	27 (0.21%)	12%	−114% to 64%	18,3%	−100.7% to 66.8%

Based on the 9 cases and 36 controls in the nested case control study, we estimated a crude VE for the influenza A(H1N1)pdm09 vaccine in preventing RT-PCR confirmed influenza was 73.3% (95%CI: 4.8%, 92.5%). The crude VE against RT-PCR confirmed influenza A(H1N1)pdm09 infection was 88% (95%CI: 25%, 98%). Due to the small sample size, no adjusted or matched analysis was performed.

### Sensitivity Analyses

In the primary analysis everyone who had received an influenza A(H1N1)pdm09 vaccine at the start of follow-up or at time of swabbing was considered exposed regardless of time since vaccination. As it takes 2 to 3 weeks to mount an immune response to seasonal influenza vaccines [11], in our primary analysis persons could have been considered exposed whilst they were not immunized. To address this potential misclassification we restricted the definition of exposure and only considered those as exposed who received a first dose more than 7 days before baseline, non-exposed were persons who were not vaccinated or vaccinated within 7 days. This decreased the crude VE against ILI to 13.3% (95%CI: −15.5%–34.9%). Only considering as exposed those who received a first dose more than 14 days before baseline and as non-exposed those not vaccinated or vaccinated within 14 days prior to baseline decreased this estimate further to 5.1% (95%CI: −36.1%, 33.8%). Restricting the analysis of the nested case control study to swabs taken 14 days after the start of vaccination resulted in a crude VE against RT-PCR confirmed influenza A infection of 17% (95%CI −563%, 90%) and a crude VE against influenza A(H1N1)pdm09 infection of 75% (95%CI: −473%, 99%).

The baseline for the cohort study was chosen relatively late (figure 2) to allow for the majority of GP-practices to have administered at least the first dose of influenza A(H1N1)pdm09 vaccine plus 14 days for the vaccine to exert its effectiveness. When applying a start of follow-up two weeks earlier (week 47 instead of week 49) the crude overall VE increased to 23% (95%CI: 4%, 38%). Applying a cut-off two weeks later (week 51 instead of week 49) decreased the crude overall VE to −7.8% (95%CI: −48.0%, 22.4%).

In a post-hoc analysis we started follow-up in October 2009 and considered exposure to A(H1N1)pdm09 vaccination to be a time dependent variable. By doing so misclassification of exposure is limited. The most noticeable increase in number of cases was seen in the ≤4 year age group. Overall, the estimates move closer towards no effect (table 4).

**Table 4:** Crude and adjusted pandemic H1N1 vaccine effectiveness per age category: Post hoc Time Dependent Analysis with baseline at 01-10-2009.

*Age Category*	*Number of ILI cases (%)*	*Crude VE*	*95%CI*	*Adjusted VE* *	*95%CI*
**Overall**		9.0%	−19.2% to 30.5%	−17.4%	−54.9% to 11.0%
**< = 4 yrs**	65 (4.6%)	−38.6%	−868.9% to 80.2%	−32.9%	−815.0% to 80.7%
**5–19 yrs**	289 (3.1%)	23.6%	−109.9% to 72.2%	34.6%	−79.8% to 76.2%
**20–49 yrs**	350 (1.5%)	21.4%	−40.2% to 55.9%	34.4%	−17.0% to 63.2%
**50–59 yrs**	154 (1.0%)	3.3%	−109.1% to 55.3%	17.3%	−79.2% to 61.8%
**60–79 yrs**	307 (0.5%)	−57.0%	−161.2% to 5.6%	−28.5%	−114.3% to 22.9%
**80+ yrs**	73 (0.6%)	5.4%	−158.2% to 65.3%	16.3%	−130.4% to 69.6%

* adjusted for number of different drugs prescribed and number of GP contacts in year before.

## Discussion

In our retrospective cohort study we found an overall small non-significant protective effect of vaccination with an MF59™-adjuvanted influenza A(H1N1)pdm09 vaccine against ILI. The VE estimates against RT-PCR confirmed influenza and A(H1N1)pdm09 infection were substantially higher, however numbers were small estimates are relatively unstable and no adjusted analysis could be performed. Limited importance should be attached to this crude estimate as it may suffer from confounding.

The adjusted VE against ILI was highest in persons between the age of 20 and 49 years (59%; 95%CI: 20%–78%) and in children between the age of 5 and 19 years (adjusted VE: 51%; 95% CI: −50% to 84%). We could not validly estimate the vaccine effectiveness in children ≤4 years as the group was very small and vaccinations could have been received through other routes than the GP. For persons between 50 and 59 years and persons between 60 and 79 years the adjusted VE was 21% (95% CI: −80%–64%), and −15% (95% CI: −90%–30%) respectively.

This is in line with findings from a large study by Castilla *et al* [5], who conducted a cohort study in children (1–17 years) and persons over 60 years, evaluating the VE of the MF59™-adjuvanted influenza A(H1N1)pdm09 vaccine against medically attended ILI. Similar to our findings, the VE in persons over 60 in their study was 25% (95%CI: −19%, 53%).

Immunosenescence resulting in reduced VE in older age groups is a known problem for seasonal inactivated influenza vaccines and adjuvants have been brought up as a possible solution [12]. As in the study by Castilla *et al* we found no evidence that the adjuvanted vaccine results into improved effectiveness against ILI in the elderly. A possible explanation of the absence of effectiveness against ILI in persons over 50 in our study is the lack of specificity of ILI for influenza, due to the presence of cross-reactive antibodies in older adults resulting from previous exposure to similar influenza strains [13]. These would protect against infection with influenza A(H1N1)pdm09 regardless of vaccination, whilst still being susceptible to a wide range of pathogens that could cause ILI. As a result ILI could be less specific for influenza in older people than in younger people who lack cross-reactive antibodies [13] leaving them vulnerable to influenza A(H1N1)pdm09 infection, hence a proportion of ILIs could be caused by influenza virus. Consequently, the specificity of ILI could not only change with time, as circulation of virus decreases, but also with age. These uncertainties underline the importance of including confirmed influenza infection as an endpoint to validate findings in the larger cohort. In our nested case control study we lacked the power to do this.

A test negative case control study evaluated the VE of the MF59™-adjuvanted influenza A(H1N1)pdm09 against laboratory confirmed influenza A(H1N1)pdm09 infection in a general population ≥10 years of age in Korea. Only14% had underlying disease. They found a VE of 73.4% (95%CI: 49.1%, 86.1%) against laboratory confirmed influenza A(H1N1)pdm09 infection [6], which did not vary significantly with age, supporting the theory that our findings are due to the lack of specificity of our endpoint rather than the vaccine. However, considering the differences in population ideally we would have validated this within our own cohort.

Several studies evaluated the effectiveness of ASO3-adjuvanted influenza A(H1N1)pdm09 vaccine against laboratory confirmed H1N1 in the general population [14], [15], [16], [17], [18], [19], reporting VE estimates between 60% and 95%. The effectiveness of AS03-adjuvanted influenza A(H1N1)pdm09 vaccine was found to be lower in an at risk population under 65 in Denmark (49% against laboratory confirmed ILI, 44% against hospitalisation) [18]. Other studies for a mix of adjuvanted and non-adjuvanted vaccines against ILI, laboratory confirmed H1N1 and hospitalisations [20], [21], [22], [23], [24] reported combined VE estimates of 52% against ILI [20], 72% to 95% against lab confirmed ILI [21], [22], [23], [24] and 90%–100% against hospitalisations [21], [23].

In our cohort, severity of underlying co-morbidity rather than its presence was a more important confounder, possibly as the majority of persons in the cohort had underlying medical conditions. The approximation used for determining severity of disease by number of different pharmaceutical compounds prescribed is a crude measure that should be further refined and validated for future influenza vaccine effectiveness studies. Also, other methods of mapping severity of underlying co-morbidity remain to be evaluated. Given the large effect of disease severity, misclassification of this covariate can be an important source of residual confounding.

Being a study using observational data, misclassification and residual confounding are a potential concern. As the likelihood of being exposed increased when moving away from the epidemic peak, and the likelihood of ILI (and the specificity of ILI to represent influenza infection) decreased away from the peak we chose the start of follow-up where the majority of vaccinated persons had received at least one dose of influenza A(H1N1)pdm09 vaccine and there was still detectable influenza transmission in the community. This had two major consequences – it limited the power of the study, and misclassification of exposure was inevitable. We evaluated the effect of time since exposure by only considering those exposed who received a first dose more than 7 or 14 days before baseline. This did have a considerable impact on the estimate of VE, decreasing it from 17.3% to 13.3% (95%CI: −15.5%, 34.9%) and 5.1% (95%CI: −36.1%, 33.8%) respectively. The reduction in VE when including the time restriction to define exposed status illustrates how misclassification of exposure dilutes the estimate in our study. As the majority of vaccinated persons had received their first dose (table 1) at start of follow-up we hope to have minimized the consequences of exposure misclassification. This was further supported by the analysis in which exposure was considered as a time dependent variable. An increase in power and shift in effect estimate was seen in children under five years, however not in other age groups indicating only limited misclassification of exposure overall.

Misclassification of exposure also may have occurred since recording of influenza vaccinations in the patient record by the GP was not compulsory and vaccinations could have been obtained through other sources. To minimize such misclassification we excluded GPs with ambiguous vaccine registration in the electronic patient record. We did miss vaccinations in children below 5, and in health care workers who received vaccinations elsewhere than at the GP. This misclassification most likely would drive the VE toward no effect.

We varied the start of follow-up to evaluate the impact of calendar time on the study. The crude VE increased to 23% (95%CI: 4%, 38%) when applying an earlier start date (week 47), and decreased to −7.8% (95%CI: −48.0%, 22.4%) when applying a later start of follow-up (week 51), illustrating that the specificity of medically attended ILI changed during the epidemic.

False-negative misclassification of ILI is likely to have occurred since people were advised to stay at home and not contact the GP with flu symptoms. Differential misclassification may have arisen if people with more serious underlying disease were more likely than other people to get the vaccination and to report ILI to their GP, leading to an underestimation of the VE.

### Conclusion

With our study we demonstrated that the approach of combining a cohort study in a primary health care database with field sampling is a feasible option to monitor VE of influenza vaccines in the future. This approach had the benefit of reliably measuring the presence of a large number of potential confounding variables, including underlying comorbidities, severity of disease, health seeking behavior, drug use patterns and vaccination history and evaluating their effect on VE estimates whilst validating the less specific outcomes that are measurable in the cohort, such as ILI, with more specific laboratory confirmed outcomes.

## References

[1] Ferguson NM, Cummings DA, Fraser C, Cajka JC, Cooley PC, et al (2006) Strategies for mitigating an influenza pandemic Nature. 442: 448–452. 10.1038/nature04795 PMC7095311 16642006

[2] Johansen K, Nicoll A, Ciancio BC, Kramarz P (2009) Pandemic influenza A(H1N1) 2009 vaccines in the European Union. Euro Surveill 14: pii: 19361. 19883538

[3] European Medicines Agency (2010) Summary of Product Characteristics for Focetria, Influenza vaccine H1N1v (surface antigen, inactivated, adjuvanted). Available: http://www.ema.europa.eu/docs/en_GB/document_library/EPAR_-_Product_Information/human/000710/WC500023749.pdf. Accessed 2013 Jan 4.

[4] Steens A, Wijnans E, Dieleman J, Sturkenboom M, van der Sande M, et al (2011) Effectiveness of a MF-59TM-adjuvanted pandemic influenza vaccine to prevent 2009 influenza A/H1N1-related hospitalisation; a matched case-control study. BMC Infect Dis 11: 196. 21767348 10.1186/1471-2334-11-196 PMC3154871

[5] Castilla J, Morán J, Martínez-Artola V, Fernández-Alonso M, Guevara M, et al (2011) Effectiveness of the monovalent influenza A(H1N1)2009 vaccine in Navarre, Spain, 2009–2010: Cohort and case-control study. Vaccine 29: 5919–5924. 21723358 10.1016/j.vaccine.2011.06.063

[6] Song JY, Cheong HJ, Heo JY, Noh JY, Choi WS, et al (2011) Effectiveness of the pandemic influenza A/H1N1 2009 monovalent vaccine in Korea. Vaccine 29: 1395–1398. 21199701 10.1016/j.vaccine.2010.12.050

[7] Vlug A, van der Lei J, Mosseveld B, van Wijk M, van der Linden P, et al (1999) Postmarketing surveillance based on electronic patient records: the IPCI project. Methods Inf Med 38: 339–344. 10805025

[8] van der Lei J, Duisterhout JS, Westerhof HP, van der Does E, Cromme PVM, et al (1993) The Introduction of Computer-based Patient Records in the Netherlands. Annals of Internal Medicine 119: 1036–1041. 8214981 10.7326/0003-4819-119-10-199311150-00011

[9] Voordouw BCG, Sturkenboom MCJM, Dieleman JP, Stijnen T, van der Lei J, et al (2006) Annual Influenza Vaccination in Community-Dwelling Elderly Individuals and the Risk of Lower Respiratory Tract Infections or Pneumonia. Arch Intern Med 166: 1980–1985. 17030831 10.1001/archinte.166.18.1980

[10] European Commission (2009) Commission Decision of 30 April 2009 amending Decision 2002/253/EC laying down case definitions for reporting communicable diseases to the Community network under Decision No 2119/98/EC of the European Parliament and of the Council. Official Journal of the European Union. Available: http://eur-lex.europa.eu/LexUriServ/LexUriServ.do?uri=OJ:L:2009:110:0058:0059:EN:PDF. Accessed 2013 Jan 4.

[11] Coordination Group for Mutual Recognition and Decentralised Procedures - Human (2009) Core SPC for trivalent influenza vaccines. Available: http://www.hma.eu/uploads/media/Core_SPCPL_Influenza_Vaccines_2008_11-Clean.pdf. Accessed 2013 Jan 4.

[12] Monto AS, Ansaldi F, Aspinall R, McElhaney JE, Montaño LF, et al (2009) Influenza control in the 21st century: Optimizing protection of older adults. Vaccine 27: 5043–5053. 19559118 10.1016/j.vaccine.2009.06.032

[13] Hancock K, Veguilla V, Lu X, Zhong W, Butler E, et al (2009) Cross-Reactive Antibody Responses to the 2009 Pandemic H1N1 Influenza Virus N Engl J Med. 361: 1945–1952. 10.1056/NEJMoa0906453 19745214

[14] Örtqvist A, Berggren I, Insulander M, de Jong B, Svenungsson B (2011) Effectiveness of an Adjuvanted Monovalent Vaccine Against the 2009 Pandemic Strain of Influenza A(H1N1)v in Stockholm County, Sweden. Clin Infect Dis 52: 1203–1211. 21507917 10.1093/cid/cir182

[15] Andrews NJ, Waight P, Yung CF, Miller E (2011) Age-specific effectiveness of an oil-in-water adjuvanted pandemic (H1N1) 2009 vaccine against confirmed infection in high risk groups in England. J Infect Dis 203: 32–39. 21148494 10.1093/infdis/jiq014 PMC3086445

[16] Skowronski DM, Janjua NZ, De Serres G, Hottes TS, Dickinson JA, et al.. (2011) Effectiveness of AS03 adjuvanted pandemic H1N1 vaccine: case-control evaluation based on sentinel surveillance system in Canada, autumn 2009. BMJ 342: doi: http://dx.doi.org/10.1136/bmj.c7297. 10.1136/bmj.c7297 PMC3033439 21292718

[17] Wichmann O, Stocker P, Poggensee G, Altmann D, Walter D, et al.. (2010) Pandemic influenza A(H1N1) 2009 breakthrough infections and estimates of vaccine effectiveness in Germany 2009–2010. Euro Surveill 15: pii: 19561. 20460094

[18] Hardelid P, Fleming DM, McMenamin J, Andrews NJ, Robertson C, et al.. (2011) Effectiveness of pandemic and seasonal influenza vaccine in preventing pandemic influenza A(H1N1)2009 infection in England and Scotland 2009–2010. Euro Surveill 16: doi:pii = 19763. 21251487

[19] Uphoff H, an der Heiden M, Schweiger B, Campe H, Beier D, et al (2011) Effectiveness of the AS03-Adjuvanted Vaccine against Pandemic Influenza Virus A/(H1N1) 2009 - A Comparison of Two Methods; Germany, 2009/10. PLoS ONE 6: e19932 doi: 10.1371/journal.pone.0019932. 21789163 10.1371/journal.pone.0019932 PMC3138735

[20] Pelat C, Falchi A, Carrat F, Mosnier A, Bonmarin I, et al (2011) Field effectiveness of pandemic and 2009–2010 seasonal vaccines against 2009–2010 A(H1N1) Influenza: estimations from surveillance data in France. PLoS ONE 6: e19621 doi: 10.1371/journal.pone.0019621. 21573005 10.1371/journal.pone.0019621 PMC3091864

[21] Simpson CR, Ritchie LD, Robertson C, Sheikh A, McMenamin J (2010) Vaccine effectiveness in pandemic influenza - primary care reporting (VIPER): an observational study to assess the effectiveness of the pandemic influenza A (H1N1)v vaccine. Health Technol Assess 14: 313–346. 20630126 10.3310/hta14340-05

[22] Valenciano M, Kissling E, Cohen JM, Oroszi B, Barret AS, et al (2011) Estimates of Pandemic Influenza Vaccine Effectiveness in Europe, 2009–2010: Results of Influenza Monitoring Vaccine Effectiveness in Europe (I-MOVE) Multicentre Case-Control Study. PLoS Med 8: e1000388 doi: 10.1371/journal.pmed.1000388. 21379316 10.1371/journal.pmed.1000388 PMC3019108

[23] Puig-Barberà J, Arnedo-Pena A, Pardo-Serrano F, Tirado-Balaguer MD, Pérez-Vilar S, et al (2010) Surveillance and Vaccine Evaluation Group during the autumn 2009 H1N1 pandemic wave in Castellón, Spain. Effectiveness of seasonal 2008–2009, 2009–2010 and pandemic vaccines, to prevent influenza hospitalizations during the autumn 2009 influenza pandemic wave in Castellón, Spain. A test-negative, hospital-based, case-control study. Vaccine 28: 7460–7467. 20875486 10.1016/j.vaccine.2010.09.042

[24] Savulescu C, Jimenez-Jorge S, de Mateo S, Pozo F, Casas I, et al (2011) Using surveillance data to estimate pandemic vaccine effectiveness against laboratory confirmed influenza A(H1N1)2009 infection: two case-control studies, Spain, season 2009–2010. BMC Public Health 11: 899 doi: 10.1186/1471-2458-11-899. 22129083 10.1186/1471-2458-11-899 PMC3262832

